# Contextual barriers and enablers to establishing an addiction-focused consultation team for hospitalized adults with opioid use disorder

**DOI:** 10.1186/s13722-024-00461-x

**Published:** 2024-04-26

**Authors:** Sandra K. Evans, Allison J. Ober, Ariella R. Korn, Alex Peltz, Peter D. Friedmann, Kimberly Page, Cristina Murray-Krezan, Sergio Huerta, Stephen J. Ryzewicz, Lina Tarhuni, Teryl K. Nuckols, Katherine E. Watkins, Itai Danovitch

**Affiliations:** 1 RAND Corporation, 1776 Main Street, 90407‑2138 Santa Monica, CA P.O. Box 2138, USA; 2 Cedars-Sinai Medical Center, 8700 Beverly Boulevard, 90048 West Hollywood, CA USA; 3 University of New Mexico Health Sciences Center, 1 University, MSC10 5550, 87133 Albuquerque, NM USA; 4 Department of Medicine, University of Massachusetts Chan Medical School-Baystate and Baystate Health, 3601 Main Street, 3rd Floor, 01107 Springfield, MA USA; 5 Departement of Medicine, University of Pittsburgh School of Medicine, 200 Meyran Ave, Suite 300, 15213 Pittsburgh, PA USA

**Keywords:** Organizational context, Consolidated framework for implementation research, Implementation science, Opioid use disorder, Medications for opioid use disorder, Addiction consult team, Collaborative care, Linkage to follow-up, Hospitalized inpatient

## Abstract

**Background:**

Hospitalization presents an opportunity to begin people with opioid use disorder (OUD) on medications for opioid use disorder (MOUD) and link them to care after discharge; regrettably, people admitted to the hospital with an underlying OUD typically do not receive MOUD and are not connected with subsequent treatment for their condition. To address this gap, we launched a multi-site randomized controlled trial to test the effectiveness of a hospital-based addiction consultation team (the Substance Use Treatment and Recovery Team (START)) consisting of an addiction medicine specialist and care manager team that provide collaborative care and a specified intervention to people with OUD during the inpatient stay. Successful implementation of new practices can be impacted by organizational context, though no previous studies have examined context prior to implementation of addiction consultation services (ACS). This study assessed pre-implementation context for implementing a specialized ACS and tailoring it accordingly.

**Methods:**

We conducted semi-structured interviews with hospital administrators, physicians, physician assistants, nurses, and social workers at the three study sites between April and August 2021 before the launch of the pragmatic trial. Using an analytical framework based on the Consolidated Framework for Implementation Research, we completed a thematic analysis of interview data to understand potential barriers or enablers and perceptions about acceptability and feasibility.

**Results:**

We interviewed 28 participants across three sites. The following themes emerged across sites: (1) START is an urgently needed model for people with OUD; (2) Intervention adaptations are recommended to meet local and cultural needs; (3) Linking people with OUD to community clinicians is a highly needed component of START; (4) It is important to engage stakeholders across departments and roles throughout implementation. Across sites, participants generally saw a need for change from usual care to support people with OUD, and thought the START was acceptable and feasible to implement. Differences among sites included tailoring the START to support the needs of varying patient populations and different perceptions of the prevalence of OUD.

**Conclusions:**

Hospitals planning to implement an ACS in the inpatient setting may wish to engage in a systematic pre-implementation contextual assessment using a similar framework to understand and address potential barriers and contextual factors that may impact implementation. Pre-implementation work can help ensure the ACS and other new practices fit within each unique hospital context.

**Supplementary Information:**

The online version contains supplementary material available at 10.1186/s13722-024-00461-x.

## Background

The inpatient hospitalization is an opportune moment to reach people with an opioid use disorder (OUD) because they may be willing to engage with treatment, including initiating MOUD, if barriers can be reduced [1–11]. However, people with OUD do not typically initiate MOUD while in the hospital or get linked with subsequent treatment for their OUD after discharge [12–14]. A growing body of research shows that addiction consultation services (ACS), which typically consist of an addiction specialist, a care manager with expertise in addiction, and sometimes other members, such as peer navigators —are an important strategy for increasing the delivery and initiation of MOUD to people with OUD during their hospitalization and linkage to outpatient care once they leave [10, 15, 16]. Some prior work suggests that hospital-based ACS are feasible and cost-effective to implement, considered acceptable to both patients and clinicians [17–24], may contribute to lower readmission rates for people with OUD [25], and increase the likelihood that patients continue MOUD post-discharge [26]. However, this prior work did not use an implementation framework or systematic process aimed at ensuring the fit of the consult team with the context-specific needs of the hospital. Additionally, few studies have used an ACS-specific intervention or used a systematic approach to identifying contextual factors that affect successful implementation, and no other studies have assessed organizational context prior to the implementation of an ACS in order to improve implementation success.

To evaluate the effectiveness of an ACS that uses a specialized intervention for improving MOUD initiation and linkage to care in hospital settings, we planned a multi-site, open label, pragmatic randomized controlled trial of an ACS with a tailored intervention called the Substance Use Treatment and Recovery Team (START) at three diverse academic hospitals in different states (California, New Mexico, Massachusetts) [27]. The primary outcomes were initiation of MOUD and linkage to OUD-focused follow-up care [27].

The START is an ACS comprised of an addiction medicine specialist (AMS) and care manager (CM) who deliver a tailored intervention based on motivational interviewing (MI) [28] and focused discharge planning [29, 30]. The START provides diagnostic assessments, makes appropriate treatment recommendations, assists with implementation of treatment plans, establishes OUD-focused discharge plans, facilitates linkage to treatment after discharge, and provides follow-up telephone calls for one month. In this pragmatic trials, each hospital’s AMS and CM were hospital staff members, and the START was integrated into the hospital workflow, relying on acceptance from the medical team for successful implementation. Further detail about the START and the study can be found in our protocol paper [27].

Understanding organizational context—particularly the perspectives of key stakeholders including clinician administrators, clinicians and patients—is an important first step for successful implementation of new practices [31]. Guided by the Consolidated Framework for Implementation Research (CFIR) [32, 33], we collected pre-implementation contextual data from key stakeholders, including hospital administrators, physicians, physician assistants, nurses, and social workers to better understand the context for implementing an addiction-focused consultation service within three diverse hospitals and to inform implementation. CFIR is an implementation determinant framework comprised of five domains that characterize the innovation or intervention, outer setting, inner setting, individuals involved, and the implementation process. Each domain contains a menu of constructs thought to affect implementation of new practices. The CFIR domains and constructs can be used to assess key determinants relevant to a given innovation. Pre-implementation contextual assessments can guide the development and tailoring of new practices and inform strategies for successful implementation. (Of note, we did not collect pre-implementation data from patients as part of this inquiry because we obtained patient input in a separate pilot study [24].)

The objective of this pre-implementation research was primarily to understand contextual factors that could affect implementation of the START for our trial, as it was directly integrated into the hospital workflow, and if successful, after the trial; we also aimed to advance the literature on contextual factors affecting hospital-based ACS more broadly. We sought to examine hospital administrator and clinician perspectives on contextual factors that could act as barriers or enablers to implementing a hospital-based ACS; assess acceptability and feasibility of an ACS team in comparison to usual care practices for people with OUD; and understand perceptions about people with OUD and the use of MOUD more generally. In this article we describe our findings and discuss how this exploration can facilitate broader implementation of hospital-based ACS for treating OUD.

## Methods

### Design

To examine potential determinants of implementation in the pre-implementation phase of the START, we conducted semi-structured interviews with hospital administrators and clinicians at each START site between April and August 2021 prior to the launch of the pragmatic trial [34]. 1 Details about the START intervention itself are described elsewhere [27]. Using a qualitative research approach, we gathered in-depth views about contextual determinants that could affect START from a range of hospital staff, including administrators, who had a unique perspective about feasibility, clinicians (doctors and nurses) who might encounter or refer patients to the START, and social workers, who might coordinate with START for discharge planning.

### Setting

Hospital sites were Baystate Medical Center (BMC) in Springfield, MA, Cedars-Sinai Medical Center (CSMC) in Los Angeles, CA, and University of New Mexico Hospital (UNMH) in Albuquerque, NM. BMC, CSMC, and UNMH are each teaching hospitals. BMC is the flagship hospital of Baystate Health, a nonprofit integrated healthcare system serving over 800,000 people in Western New England and providing care for approximately 45,000 inpatients, 197,000 emergency care patients, and 1.8 million outpatient visits annually [35]. CSMC is a nonprofit academic healthcare organization serving more than 1 million people each year in the diverse Los Angeles community and beyond [36]. UNMH is part of the UNM Medical Group that includes 30 clinics and six hospitals and medical centers across the state, collectively providing care for 900,000 outpatient visits, 22,000 surgical cases, and 100,000 emergency room visits annually [37].

In both Los Angeles County, CA and Bernalillo County, NM (home to CSMC and UNMH, respectively) approximately half of the population is Hispanic or Latino, whereas about a quarter of residents in Hampden County, MA (home to BMC) have a Hispanic or Latino background. Bernalillo County has the greatest proportion of American Indian and Alaska Native residents (4.9% vs. <1.0% in Los Angeles and Hampden counties). Los Angeles County has the greatest proportion of Asian residents (14.8% vs. approximately 2.6% in Bernalillo and Hampden counties), foreign-born residents (33.5% vs. 9.2–10.2%), and those who speak a language other than English at home (55.8% vs. 26.2–27.5%) [38–40].

### Data collection processes

#### Sampling and recruitment

We used a mix of purposive and snowball sampling to develop a sample of interviewees at each hospital. Hundreds of employees work at each participating hospital and potentially eligible patients for the START intervention could be admitted to the hospital in any unit. Therefore, we could not simply select a sample of clinicians from particular hospital units. Instead, we asked START researchers who were also clinicians and addiction medicine specialists at the participating hospitals to provide a list of possible interviewees representing different roles, including administrative leadership, attending physicians, nurses, and social workers. Our sampling approach prioritized staff members who were most likely to interact with potential START participants. The START clinicians sent an email to potential participants to say that START research personnel would be reaching out to request their participation in an interview. START researchers then emailed each potential participant (up to three times) to request and schedule interviews.

We did not have a target number of interviewees, particularly since we were seeking additional interviews via snowball sampling. Lists of possible interviewees ranged from 12 to 20 individuals. After each interview, we asked participants to recommend key individuals to interview whose insights could assist with the implementation of the START.

#### Recruitment

The protocol was reviewed and approved by the Cedars Sinai Medical Center Institutional Review Board (IRB). Cedars Sinai served as the IRB of record. BMC and UNM ceded IRB review to Cedars-Sinai via a SMART IRB agreement; the respective IRBs at BMC and UNM reviewed for local context only. Approval included a waiver of written informed consent for participants, given low risk. While the identities of interviewees were visible to the researchers who conducted interviews, all potentially identifying information has been kept confidential in the reporting of results.

#### Data collection

Three team members with experience in semi-structured interviewing and qualitative analysis conducted the semi-structured interviews via the Microsoft Teams videoconferencing platform. Each interview took approximately 30–45 min to complete. The interview guide consisted of open-ended questions to engage participants in discussion about their professional views and experiences. The interview guide was informed by CFIR categories and related materials including the CFIR qualitative interview guide tool [41]. We used these resources to develop interview questions that both addressed the context of the START and the most relevant components of the CFIR. The resulting interview guide addressed the following domains (see Additional File 1):

- Acceptability of addressing OUD during the inpatient stay and MOUD (focus on usual care).
- Perceptions about the START.
  - *CFIR constructs addressed*: Relative Advantage and Tension for Change; Compatibility; Clinician/Hospital Needs and Resources; Linking.
- Patient and organizational needs.
  - *CFIR constructs addressed*: Patient Needs and Resources; Adaptability and Culture.
- Enabling factors.
  - *CFIR constructs addressed*: Stakeholders and Opinion Leaders; Communication.
- Possible additional barriers.

We pilot-tested the interview guide with members of the START steering committee who were knowledgeable about both the START and the sites that were involved. These pilot interviews allowed us to refine question wording and order, as well as content.

Because interviews were conducted in the pre-implementation phase, the guide featured prospectively framed questions, for example, about whether participants could foresee any barriers or challenges related to how the START would fit into their workflow. Interviews were recorded and transcribed with participants’ verbal consent. As an incentive, each interviewee was offered a $50 gift card for their participation.

### Qualitative analysis

We conducted thematic analysis to examine themes that cut across the interviews [42, 43]. Taking a primarily deductive approach, we developed a codebook structured around the topics from the interview guide and related CFIR constructs [32]. In addition to referencing the 2009 CFIR constructs that informed the interview guide, we referred to CFIR resources including the CFIR codebook to inform our analysis [44]. Through extensive discussions among our interview team and with input from the broader research team involved with the START, we narrowed down, and in some cases tailored or combined, the CFIR constructs that were most relevant to the context of our data and research objectives to include: (1) Outer Setting constructs, Patient Needs and Resources, Linking (adapted from Cosmopolitanism); (2) Combined Intervention and Inner Setting constructs, Relative Advantage and Tension for change, and Adaptability and Culture; (3) Inner Setting constructs, Clinician/Hospital Needs and Resources (adapted from Implementation Climate), Communication (adapted from Networks and Communications), and Compatibility; and (4) a combined Process construct, Key Stakeholders (adapted from Opinion Leaders and Formally Appointed Internal Implementation Leaders) (see Table 1).

**Table 1:** Codebook summary by CFIR domain

#	CFIR construct/code	Description^a^
*CFIR domain: outer setting*		
1	Patient needs and resources	This outer setting characteristic refers to “the extent to which patient needs, as well as barriers and facilitators to meeting those needs, are recognized and prioritized by the organization” in usual care or by the START
2	Linking	Linking is adapted from the Outer Setting code, “Cosmopolitanism” meaning “the degree to which an organization is” or can be “networked with other external organizations.” We adapted this code because of specific features of the START including connecting PWOUD with community clinicians
*CFIR domain: intervention characteristics and inner setting*		
3	Relative advantage and tension for change	Relative advantage is an intervention characteristic meaning “stakeholders’ perception of the advantage of implementing the intervention versus an alternative solution.” Tension for change is an inner setting characteristic describing “the degree to which stakeholders perceive the current situation as intolerable or needing change.” Although these codes reflect a mix of intervention/innovation and Inner setting constructs, in the context of the START they are used to address comments that reflect the extent to which there is a sense of urgency or need for the intervention
4	Adaptability and culture	Adaptability is an intervention characteristic on “the degree to which an innovation can be adapted, tailored, refined, or reinvented to meet local needs.” Culture is an Inner Setting characteristic describing the “norms, values, and basic assumptions of a given organization” Although these codes stem from intervention and inner setting domains, they reflect comments that capture the extent to which the intervention may fit different subcultures (including both organizational and patient-oriented cultures) within the hospital setting
*CFIR domain: inner setting*		
5	Clinician/hospital needs and resources	This code is adapted from the inner setting characteristic, implementation climate meaning “The absorptive capacity for change, shared receptivity of involved individuals to an intervention, and the extent to which use of that intervention will be rewarded, supported, and expected within their organization.” Comments focus on organizational topics including clinician experience and acceptability. This code tends to focus on clinicians’ points of view (versus perspectives of people with OUD)
6	Communication	This inner setting code is based on the CFIR characteristic “networks and communications” meaning “the nature and quality of webs of social networks,” and “formal and informal communications” at each site
7	Compatibility	This inner setting characteristic means “the degree of tangible fit between meaning and values attached to the intervention by involved individuals, how those align with individuals’ own norms, values, and perceived risks and needs, and how the intervention fits with existing workflows and systems.”
*CFIR domain: process*		
8	Key stakeholders	Two CFIR categories were used to define the stakeholders code. Opinion Leaders is a Process code pertaining to “individuals in an organization that have formal or informal influence on the attitudes and beliefs of their colleagues with respect to implementing the innovation.” We also included the process code, “formally appointed internal implementation leaders,” here to address comments about “individuals from within the organization that are directly impacted by the innovation, e.g., staff responsible for making referrals to a new program or using a new work process.”
*Sorting codes*: codes that are used for double- or m1-8 to allow analysis to explore topics by code category (e.g., “linking” barriers vs. facilitators)		
9	Barrier	Excerpts that describe a barrier or constraint. Can include barriers related to either usual care or the implementation of the START intervention
10	Recommendation, facilitator, or idea	Recommendation, facilitator, idea, or suggestion about how to deal with a barrier or constraint. Can include recommendations related to either usual care or the implementation of the START intervention
11	COVID	Comments related to COVID; for example, how COVID has affected services and how it could affect the START intervention

^a^ Codes adapted from the CFIR. Quotes used in the descriptive text stem from the CFIR Guide website [48]

We then adapted these categories and their definitions through further discussion and practice application to our interview data. We also included three additional codes to help us sort the data based on reported barriers, recommendations or ideas for the START intervention, and impacts of the COVID-19 pandemic (e.g., the study was launching relatively early in the pandemic when normal hospital protocols were set aside to cope with the influx of COVID patients and to mitigate risks related to potential COVID exposure). These sorting codes helped us more easily examine combinations of codes such as barriers related to the CFIR-derived code, Adaptability and Culture. More than one code could be applied to any given excerpt (codes were not mutually exclusive). A summary of our codebook is provided in Table 1.

Three members of the research team coded interview data in Dedoose [45]. We took several iterative steps to train the coders and assess intercoder reliability. First, we practice-coded two to three transcripts and updated the codebook by refining codes, their descriptions, and examples where necessary. We then triple-coded the same sample of five transcripts to measure intercoder reliability. This sample included transcripts from each site. Each member of the coding team coded the same excerpts from the five sample interviews independently. We calculated Krippendorff’s Alpha to evaluate the consistency of code application by the three coders [46]. For the codes that scored lower than 0.70, the coders discussed discrepancies at length in order to further refine the codebook and to make sure coders were consistent in how we were interpreting and applying codes. We then divided up the transcripts among the three coders and coded these samples in Dedoose.

## Results

We interviewed 28 staff members from the three hospitals participating in the START study. Table 2 reports the number of participants by site and role, in addition to the response rates for each site and overall (52.8%). Across sites, the most common interviewee role was attending physician (*n* = 10), followed by social worker (*n* = 7), administrative leader (*n* = 6), and nurse (*n* = 5). Table 2 lists participants by site (we combined administrative leaders and attending physicians in Table 2 to ensure confidentiality for participants; at least one administrator and one attending were interviewed at each site).

**Table 2:** Number of participants by hospital site and role

Site	Administrative leaders & attending physicians	Nurse	Social worker	Total	Response rate
Baystate Medical Center	4	0	2	6	6/15 (40.0%)
Cedars-Sinai Medical Center	6	2	3	11	11/17 (64.7%)
University of New Mexico Hospital	6	3	2	11	11/21 (52.4%)
Total	16	5	7	28	28/53 (52.8%)

In the following section we summarize the overarching themes from the analysis of the interview data. We also describe examples of practical advice provided to research team to help address potential barriers in the pre-implementation period. We grouped the overarching themes into four categories:

- **Urgency for Change**: There is a sense of urgency for change as well as perceived advantages and compatibility with current practices of an ACS. This included results from the Outer Setting code “Relative Advantage/Tension for Change” and Inner Setting codes “Compatibility” and “Clinician/Hospital Needs and Resources”.
- **Culturally Appropriate Adaptations**: Intervention adaptations are necessary to meet local and cultural needs. This included results from the Intervention code “Adaptability/Culture”.
- **Post-discharge Linkage**: Linking to other clinicians and organizations is needed to support OUD care. This included results from the Outer Setting codes “Linking” and “Patient Needs and Resources.”
- **Opinion Leader Engagement**: It is important to engage key stakeholders across departments and roles. This included results from the Inner Setting code “Communication” and the Process code “Opinion Leader/Stakeholder Engagement.”

Because codes used in the qualitative analysis process were not considered to be mutually exclusive, we found thematic overlap across certain CFIR-based codes. We therefore grouped findings under similar overarching themes. We also examined code co-occurrence data within Dedoose to check the extent to which codes were double-coded as an additional step to validate the extent to which findings in certain categories tended to overlap.

- ***Urgency for Change. There is a sense of urgency for change as well as perceived advantages and compatibility with current practices of an ACS.***

An innovation is unlikely to succeed unless those involved see a need for it and perceive it as fitting with existing norms, values, and workflows [47]. Most interviewees across sites agreed that there was a need for change from usual care to meet the needs of people with OUD, and the START seemed to offer an advantage. For example, an administrative leader from CSMC noted a critical gap in care that an ACS team could help fill:

“*I think there’s a lot of frustration for our social work team and for our clinicians. You get this person out of an and the resulting actions taken in response’s a cry for help. You get them stabilized and then to throw them out the door and not give them any support, it feels wrong. It feels like we’re abandoning them.*” (Administrator, CSMC).

Some interviewees also discussed how the teaching hospital culture and focus on education aligns with openness to adopt new programs like addiction-focused consult liaison services. A social worker from UNMH noted:

“*I feel like we’re actually fairly open to new programs, new ideas, because a lot of what we, there’s a lot of research going on at UNM, a lot of new trials for, not just this, but lots of different areas and I feel like, because we’re a teaching institution, it’s pretty common for new practices to come up. I think, especially in an area like this that is so needed, I think people will be open to some of these changes, whatever they end up being, so I don’t think it’s uncommon for us to go through new policies or practices like this, if it’s going to, in the long run, benefit our patients and the hospital and New Mexico.*” (Social worker, UNMH).

At BMC, interviewees discussed how aspects of usual care fell short in comparison to what the START proposed. During the previous year, BMC initiated an ACS to address infrequent initiation of MOUD. The ACS consisted of an addiction medicine specialist, but it did not have a care manager or pre-specified intervention. Although the ACS was an improvement over their earlier usual care approach in that it started to improve MOUD initiation, it still faced limitations. The new ACS was composed of a single addiction medicine specialist with no care manager or post-discharge planning intervention. Further, even with the new ACS, there was still no opportunity to actively link patients to OUD treatment or support them after discharge. The ACS offered through START augmented existing services by adding a discharge planning intervention and a care manager to implement it and provide active post-discharge linkage and support.

A CSMC social worker noted that addiction medicine is its own specialty and not their area so this type of team would be welcome:

“*I would be a fan of the resource because… some of the patient population that are falling under the OUD -- they may have mental health illness in addition to some of their other medical conditions, and so it is really, really difficult when you have those other issues as well. The more specialized resources that we have to better care for our patients, it’s advantageous for us. I think there’s going to be lots of advantages that can really maybe open some doors for some people that we didn’t know existed.*” (Social worker, CSMC).

Some CSMC interviewees also discussed how certain concerns among people with OUD, such as willingness to go to treatment and challenges with pain management, such as the perception among clinicians and patients that treatment with MOUD may conflict with the need for pain management, may deter the perceived advantages and compatibility of an ACS offered through the START. A CSMC interviewee also noted the challenge of selecting people with OUD that would qualify for the intervention, including the concern that some individuals may not be in the hospital long enough to be identified and referred to the START.

There were some differences between the hospitals with regard to Tension for Change and Relative Advantage of the START over existing programs. As noted above, BMC had already started a consultation service to address the high need for OUD treatment, but the existing service was perceived as “stretched thin” and in need of a care manager. Perception of need at UNMH also was high, with many clinicians noting the need for trained social workers to facilitate linkage to community programs. UNMH personnel also discussed that while there was not an existing ACS, the idea had been discussed among leadership and clinicians and there was general agreement about the strong need, but the hospital had not yet implemented one. As this UNMH nurse noted to emphasize the need,

*I would say [OUD care] is very important. In New Mexico, we have a long-standing issue with multi-substance abuse, opioid abuse…We’re the academic medical center, but we’re also the public hospital for Bernalillo County and the City of Albuquerque, which is the largest metropolitan Center in New Mexico so we see many patients with substance abuse disorders.* (Nurse, UNMH)

At CSMC, based on comments made during the interviews indicating lack of certainty about the number of people with OUD passing through the hospital, the perception of need for START did not seem to be as strong as at BMC or UNMH, where participants saw OUD as more prevalent. However, CSMC clinicians did see a need for START because of lack of expertise to prescribe MOUD on the medical team and to support patients after discharge.

- ***Culturally Appropriate Adaptations. Intervention adaptations are necessary to meet local and cultural needs.***

Interviewees across sites discussed the extent to which the START intervention should be tailored for various groups of people with OUD based on economic stability factors, culture, and language. For example, a social worker at BMC noted:

“*I think just that so many of our patients here are extremely limited, like, socio-economically, so there are going to be tons of patients who just truly can’t get anywhere where they would need to go for treatment. Or just so many constraints like that or no supports once they leave here.*” (Social worker, BMC).

Similarly, as discussed by an attending physician at CSMS, low-income populations may have trouble linking to follow-up care:

“*We take care of often uninsured, underinsured, unrepresented, undocumented patients and there are, just as many issues with opioid disorder in that population of patients, if not more, but they have very little to no follow up at all on discharge.*” (Attending physician, CSMC).

Interviewees across sites emphasized the desire for linguistic and cultural patient-clinician concordance to improve patients’ access to services and heighten the intervention’s impact among diverse patient populations. Having team members who share or are familiar with patients’ cultural backgrounds would help develop trusting relationships with patients.

Because the sites are located in dissimilar locations, there was some nuance in how participants suggested how adaptability could be valuable to the START. For example, participants from BMC focused primarily on the need to support low socioeconomic status patients in addition to patients with different cultural and linguistic backgrounds. CSMC’s population was unique in its wide range of patients across the socioeconomic spectrum from the unhoused to the very wealthy, in addition to patients with different cultural and linguistic backgrounds. UNMH participants emphasized the importance of working with members of Native American populations and underserved populations, including patients who had to travel very long distances to access care at the hospital. As one participant noted, *“We [have] a large Native American population state and that’s a core part of our identity as a hospital…And a lot of our patients don’t have very many resources at all [so] understanding that is part of what we do”* (Administrator, UNMH).

- ***Post-discharge Linkage. Linking to other clinicians and organizations is needed to support OUD care.***

At all three sites, participants noted the challenge of referring people with OUD to acute care facilities after they start taking MOUD. Post-discharge linkage was seen as an essential component of patient care—dependent not only on logistics connecting with outpatient facilities that offer medication therapies for OUD, but also on the support that can begin in the hospital.

The lack of a systematic way of transitioning patients to outpatient care was seen as a major barrier and not a standard practice. A CSMC physician noted that “*there is not a robust outpatient pain management network of physicians*” and an administrator at UNMH similarly perceived limited access to outpatient specialty treatment for people with OUD:

“*We have very little access for the patients that come into the hospital or come into the emergency department to establish them with follow up to continue treatment should we initiate it in the, in either the inpatient or emergency department setting. So, it’s a tremendous problem. We see it all the time and it’s hard to know what to do with it. Because when people leave the hospital, there are so few ways in which, we can ensure a transition of care.*” (Administrator, UNMH).

Interviewees also discussed that fact that few acute-care rehabilitation programs would accept patients who have already started MOUD. This issue was particularly prevalent at UNMH, with almost all interviewees noting linkage barriers due to regulations preventing skilled nursing facilities (SNFs) from administering or treating patients who are on MOUD. Clinicians described how treatment planning for people with OUD often depends on where they need to receive care after discharge. This can be a conundrum, because people with OUD, who likely cannot be released home with peripherally inserted central catheter (PICC) line and intravenous (IV) antibiotics, generally are the ones who need to go to a SNF. For example, a nurse at UNMH noted:

“*Our patients with opioid use disorders oftentimes, how we address it and treat it often depends on what we think their discharge plan is going to be. So, a very common scenario is that we have a person with opioid use disorder, IV drug use in particular, who comes in for things like endocarditis and bacteremia, and has to complete long-term antibiotics. In New Mexico or in Albuquerque there’s basically maybe two skilled nursing facilities that will take somebody on say, methadone or buprenorphine*.” (Nurse, UNMH).

Clinicians at BMC similarly described how resources for mental health services are limited in the Springfield, MA area when compared to the eastern part of the state toward Boston.

Additionally, building relationships with people with OUD and meeting them where they are was seen as critical in shepherding individuals from the inpatient to outpatient setting. Participants noted that linkage can vary in terms of providing resources versus connecting patients with an external facility, and that more substantial linkage efforts prior to discharge reduces the burden on the patient for seeking and obtaining necessary care.

Lastly, given the context of COVID-19 or future public health emergency, linking to outpatient care could prove to be more difficult. A social worker at UNMH described such difficulties for patients experiencing homelessness:

“*I know we had more post-discharge issues during COVID, just even a harder time linking people to community clinics for medication assisted treatment, just because…a lot of places closed down significantly for a lot of homeless patients. They were staying at, you know, city funded motels and trying to get their medications delivered there, especially for patients on methadone every day was a significant barrier to discharging people…We were talking to most patients over the phone in a room, so that was really difficult just from a communication perspective.*” (Social worker, UNMH).

A distinct difference between sites regarding linkage is that clinicians at BMC were very concerned about lack of available community treatment and social services for patients, while at CSMC the concern was less around community availability of services and more about ensuring patients get linked to them. One clinician at CMSC discussed the need for a “warm handoff” to follow-up clinicians or treatment programs, while another suggested the need for telehealth for follow-up because of the size of the county and services being located far from where patients live.

- ***Stakeholder Engagement. It is important to engage key stakeholders across departments and roles.***

Interviewees across sites discussed the diversity of actors involved in care for people with OUD that would be critical for the success of the ACS team intervention, including but not limited to hospital administrators; department leaders; social workers; case managers; physicians (e.g., psychiatrists and internal medicine specialists); and nurses. Interviewees discussed how key stakeholders span disciplines and teams. One interviewee from CSMC noted that key stakeholders would include the:

*“…Pain management team, definitely psychiatry team and I think social services because resources are necessary to sustain any kind of a program. We need social services to find the right resource for them, when they are no longer in our supervision*.” (Nurse, CSMC).

Similarly, an interviewee noted that:

“…*for champions, I actually think the unit nursing staff can be really helpful, right, beyond leadership, because I think they’re there all the time, right and can really influence whether or not, at least in my experience, whether or not this program will float or not.*” (Attending physician, UNMH).

Interviewees also discussed that, not only will stakeholder involvement aid in the implementation of the intervention, but having important members of the hospital involved and supportive of the intervention will be essential in funding these types of programs following the study. An attending physician at BMC noted:

*“The big thing for making sure that the program is adequately funded and staffed. I think they need to have a lot of support from our patient safety and quality department…I think the high-level c-suite people do believe that it’s important. I think it’s just a matter of justifying the cost to compared to all the other things we have to pay for.*” (Attending physician, BMC).

Receiving support from specific programs such as pain management, psychiatry, and other social services was also thought to be necessary to sustain this type of intervention in the long term. There were very few differences among the sites with regard to this theme. Overall, participants emphasized the need to engage with different types of department leaders and to spread awareness to stakeholders, including physicians, through effective communication.

### Implications for research study implementation

Because this pre-implementation research also had practical, near-term implications for the research study, we discussed preliminary findings and their implications with the research team in August of 2021 prior to the fall launch of the START, and developed actions to address implementation issues. Table 3 summarizes those recommendations and the resulting actions taken in response.

**Table 3:** Study-informed START recommendations and implementation actions

Recommendation	Implementation actions
*Urgency for change*	
• More education about MOUD for providers could help raise awareness; residents, in particular, may be interested • Pay attention to the availability and workload of the START during the study; demand may be high	• Lower perception of urgency or need for the START to address opioid use disorder at CSMC led to hospital-wide education about the need for and purpose of the START. This included Grand Rounds and other hospital presentations, led by the study PI
*Culturally appropriate adaptations*	
• START team should be prepared to navigate common patient barriers (e.g., stages of readiness, lack of social support, socioeconomic challenges like being under- or uninsured, lack of regular access to a phone, unhoused, etc.)	• At all hospitals, the need to address cultural and linguistic diversity was met through some adaptations to language and goals of the START • At UNMH, the need for deep cultural understanding of the Native American culture was met by a care manager with decades of experience working in Albuquerque with this population
*Post-discharge linkage*	
• Detailed information about specific community provider services and their insurance policies will probably be useful to the care managers • Make sure referral list is updated regularly to reflect changes in community service settings • Over-communicate/demonstrate to medical staff that the START does not delay patient discharge by design • Referral resources can be updated by care managers with additional, nuanced linkage/referral tips	• START CMs either already had or developed relationships with outpatient providers in each community to facilitation linkage • At BMC and CSMC, the START addressed need for improved linkage through the 1-month of follow-up calls by the CM that already were part of the intervention • At UNMH in particular, where linkage of OUD patients on MOUD to acute care facilities was typically impeded, the AMS and CM worked to change hospital and acute care facility policy and practice to allow for this transition
*Opinion leader engagement*	
• Provide hospital-wide communication about the START including who is involved and how to refer patients • Ensure that medical teams are aware of the START through Dear Doctor letter, FAQs, other site-specific methods • Provide multiple ways to contact the START (e.g., via paging systems, EMR, texting app, weekly team meetings, etc.)	• All hospitals conducted hospital-wide outreach to inform departments about the START. This included presentations and grand rounds, emails to department heads, and a flyer posted in break rooms

Many action items for sites related to communication among hospital providers and staff, for example, to leverage CSMC Grand Rounds as a venue to educate medical teams about OUD prevalence and the START intervention. Interviewees across all sites recommended actions to reduce barriers to linking hospitalized patients with OUD to post-discharge care. Care managers at UNMH, for example, could help facilitate the linking process by updating referral resources with tips specific to the local context. We also produced an FAQs document about the START that could be used or adapted at each site to help support hospital-wide communication about the START. The FAQ document was based on common questions from interviewees, such as how to contact representatives from the START or how the START was designed to not impact patient length of stay.

## Discussion

This qualitative study explored perspectives on contextual barriers and enablers to implementation of an ACS and tailored intervention called the START to improve MOUD initiation and linkage to services for hospitalized people with OUD. Our thematic analysis pointed to several common findings across the three disparate sites. Overall, participants saw a strong need for change from usual care to provide care for people with OUD and comments tended toward viewing the START as an acceptable and feasible option. Additionally, the START was considered likely to be compatible with current practices in that it could easily fit into medical team workflows. Shared concerns across sites included the need to adapt the START to meet local, cultural and linguistic needs such as the ability to meet the needs of very low-income individuals as well as those from diverse racial and ethnic groups and the need for hospital-wide information about the need for START and how it would fit into the workflow. Linking patients to community clinicians for post-discharge care was also described as a potential impediment to the success of the ACS.

While there were relatively few differences in perspectives among the three sites, some of these differences were notable. Most differences had to do with how the START should be tailored to meet the needs of specific patient populations. Additionally, perceptions about OUD prevalence varied in that at one site in particular (CSMC), where several interviewees recommended greater educational opportunities for medical teams to learn about the prevalence and treatment of OUD. Further, there were perceived differences in current gaps in care, such as the need for a care manager to complement an existing addiction medicine specialist to support linkage to community clinicians, versus concerns about availability of community clinicians and limitations of SNFs for people on MOUD. These types of differences about tailoring, perceptions about the degree to which OUD is prevalent in the patient population, and differences in gaps in care are likely to be relevant to other sites attempting an intervention.

Strengths of this study included the diversity of perspectives gathered across three different geographic areas (CA, MA, NM) and roles within participating hospital sites, including clinicians (social workers, physicians, nurses) and administrative leadership. Additionally, the CFIR was used to analyze contextual factors in a way that could help inform the implementation of similar ACS in other hospital settings. For example, other sites may consider engaging with stakeholders via interviews or other stakeholder discussion forums using CFIR categories like those used in this study to help introduce or preview the implementation of a given initiative and to tailor the ACS to meet identified barriers. By understanding contextual factors such as perceptions about compatibility and feasibility, adaptability, and stakeholder views, an innovation may be more likely to avoid at least some barriers in implementation. Finally, collecting and sharing pre-implementation feedback allowed the START implementation team to adapt the intervention accordingly.

Several limitations should be considered. First, interviews were conducted in Spring and Summer of 2021 during the COVID-19 pandemic, which may have impacted participation in the study. At UNMH in particular, staff shortages were a widespread concern which could have limited participation. Second, there is the potential for selection bias; that is, that the sample is biased toward staff and clinicians at the forefront of OUD care because they were referred by study team members at each hospital. To try to avoid this possibility, we let the study team members who recommended participants know that we were hoping to hear a range of views, even from those who might not encounter or support the START. Third, because patients can enter a hospital in multiple ways and see many clinicians, it would have been impossible to speak with every clinician that could come in contact with a potential START patient at each site. Knowing this limitation, we considered interviewing a purposively selected sample to be the most practical method to gather in-depth data in the timeframe available for this project.

Future research could examine post-implementation interviews with hospital staff and clinicians that came into contact with the START during the study period to compare differences in perspectives and perceptions of needs. This approach would provide an opportunity to compare pre- and post-implementation perceptions about the START. It would also provide a way to examine the START as a program that was implemented during the COVID-19 pandemic and that continued into the post-pandemic period (i.e., when COVID was still present but no longer considered a government emergency in the U.S.). Additionally, researchers could explore how the CFIR constructs explored in this research relate to START implementation outcomes with the goal of identifying drivers that impact implementation. Further, additional, qualitative data collection from patient participants in the START study could be useful for understanding patient experiences with the intervention as well as with their experiences linking to post-discharge follow-up care.

In sum, this qualitative pre-implementation work is an approach other studies can use to preview potential barriers and contextual factors that may impact the implementation of ACS and has broader implications for implementing and sustaining ACS and other new practices within the inpatient setting.

### Electronic Supplementary Material

Below is the link to the electronic supplementary material.

Supplementary Material 1

## Data Availability

The deidentified qualitative data generated and analyzed during this study can be made available from the corresponding author on reasonable request and with execution of appropriate Data Use Agreements.

## Footnotes

1 Implementation is typically broken out into phases including pre-, during, and post- because these time periods require different steps for a given intervention. In this research we refer to the pre-implementation phase as the time period prior to the launch of the START in which researchers were planning and working with participating hospitals to lay the foundation for the intervention.

## Abbreviations

ACS: addiction consultation services
BMC: Baystate Medical Center
CFIR: Consolidated Framework for Implementation Research
CSMC: Cedars-Sinai Medical Center
MOUD: medications for opioid use disorder
OUD: opioid use disorder
SNF: skilled nursing facilities
START: Substance Use Treatment and Recovery Team
UNMH: University of New Mexico Hospital
