# Supplementary material for: Contextual barriers and enablers to establishing an addiction-focused consultation team for hospitalized adults with opioid use disorder
Source: Addict Sci Clin Pract. 2024 Apr 26;19:31. doi: 10.1186/s13722-024-00461-x (PMC11046820; doi:10.1186/s13722-024-00461-x)
Supplement: Supplementary file 1 — Supplementary Material 1 [file 13722_2024_461_MOESM1_ESM.docx]

**Supplementary Material:** Interview Guide

| **Topic Category** |  | **Interview Question** |
| --- | --- | --- |
| Acceptability of Addressing OUD during the Inpatient Stay & MOUD (focus on usual care) | 1. | [Physicians and Social Workers only] How frequently do you come across patients with an opioid use disorder? |
|  | 2. | [Physicians and Social Workers only] If a diagnosis of opioid use disorder is not already in a patient's chart, how is it typically determined that a patient has an opioid use disorder? |
|  | 3. | [Physicians only] About how frequently, if ever, do you prescribe medication to treat opioid use disorder while patients are still in the hospital? I'm referring to initiating medication that they can continue after they leave the hospital, not only to take while they are in the hospital. |
|  | 4. | [Physicians only] What are some of the barriers to addressing a patient’s opioid use during their inpatient stay? What about barriers to linking them to treatment after? |
|  | 5. | [Administrators only] How important is substance use disorder care in general, and opioid use disorder care specifically, to the institution? |
|  | 6. | [Administrators only] Is substance use/opioid use disorder care part of the mission at [your hospital]? |
| Perceptions about the START  *CFIR constructs addressed:* Relative Advantage and Tension for Change; Compatibility; Provider/Hospital Needs and Resources; Linking | 7. | Do you think there is a strong need for the START at this hospital? |
|  | 8. | How does the START compare to other similar existing programs in your setting to help patients with opioid use disorder, if there are other programs? |
|  | 9. | How effective do you think START would be in helping people with opioid use disorder? |
|  | 10. | How well do you think START would fit into the inpatient workflow? |
|  | 11. | What is your best guess about how other [physicians, staff members, administrators] would feel about working with START? |
| Patient and organizational needs  *CFIR constructs addressed:* Patient Needs and Resources; Adaptability and Culture | 12. | How do you think patients here will respond to working with the START? |
|  | 13. | How could the START be changed to be more sensitive to the background or culture of patients in your hospital? |
| Enabling factors  *CFIR constructs addressed:* Stakeholders and Opinion Leaders; Communication | 14. | Who are the key influential individuals at the hospital who should get on board with the START to help it be successful? |
|  | 15. | What usually determines whether new treatments are used by providers at this hospital? |
| Possible barriers, if not already discussed: | 16. | What barriers might you face in working with the START team to help patients with opioid use disorders? |
|  | 17. | How do you think COVID-related changes to your routine, or changes to the hospital more broadly, may impact the START intervention? |
